# Predictive metabolites for incident myocardial infarction: a two-step meta-analysis of individual patient data from six cohorts comprising 7897 individuals from the COnsortium of METabolomics Studies

**DOI:** 10.1093/cvr/cvad147

**Published:** 2023-09-14

**Authors:** Ana Nogal, Taryn Alkis, Yura Lee, Domagoj Kifer, Jie Hu, Rachel A Murphy, Zhe Huang, Rui Wang-Sattler, Gabi Kastenmüler, Birgit Linkohr, Clara Barrios, Marta Crespo, Christian Gieger, Annette Peters, Jackie Price, Kathryn M Rexrode, Bing Yu, Cristina Menni

**Affiliations:** Department of Twin Research, King’s College London, St Thomas’ Hospital Campus, Westminster Bridge Road, SE1 7EH London, UK; Department of Epidemiology, Human Genetics and Environmental Sciences, University of Texas Health Science Center at Houston School of Public Health, 1200 Pressler St, Suite E407, Houston, 77030 TX, USA; Department of Epidemiology, Human Genetics and Environmental Sciences, University of Texas Health Science Center at Houston School of Public Health, 1200 Pressler St, Suite E407, Houston, 77030 TX, USA; Faculty of Pharmacy and Biochemistry, University of Zagreb, Zagreb, Croatia; Division of Women’s Health, Department of Medicine, Brigham and Women’s Hospital, Boston, MA, USA; Faculty of Medicine, University of British Columbia, Vancouver, BC, Canada; Cancer Control Research, BC Cancer, Vancouver, BC, Canada; Usher Institute of Population Health Sciences and Informatics, University of Edinburgh, Edinburgh, UK; Research Unit of Molecular Epidemiology, Helmholtz Zentrum München, Neuherberg, Germany; Institute of Bioinformatics and Systems Biology, Helmholtz Zentrum München, Neuherberg, Germany; Institute of Epidemiology, Helmholtz Zentrum München, Neuherberg, Germany; Department of Nephrology, Hospital del Mar, Institut Hospital del Mar d´Investigacions Mediques, Barcelona, Spain; Department of Nephrology, Hospital del Mar, Institut Hospital del Mar d´Investigacions Mediques, Barcelona, Spain; Research Unit of Molecular Epidemiology, Helmholtz Zentrum München, Neuherberg, Germany; Institute of Epidemiology, Helmholtz Zentrum München, Neuherberg, Germany; Usher Institute of Population Health Sciences and Informatics, University of Edinburgh, Edinburgh, UK; Division of Women’s Health, Department of Medicine, Brigham and Women’s Hospital, Boston, MA, USA; Department of Epidemiology, Human Genetics and Environmental Sciences, University of Texas Health Science Center at Houston School of Public Health, 1200 Pressler St, Suite E407, Houston, 77030 TX, USA; Department of Twin Research, King’s College London, St Thomas’ Hospital Campus, Westminster Bridge Road, SE1 7EH London, UK

**Keywords:** Myocardial infarction, Metabolomics, Biomarkers, Two-step individual patient data meta-analysis, Amino acids

## Abstract

**Aims:**

Myocardial infarction (MI) is a major cause of death and disability worldwide. Most metabolomics studies investigating metabolites predicting MI are limited by the participant number and/or the demographic diversity. We sought to identify biomarkers of incident MI in the COnsortium of METabolomics Studies.

**Methods and results:**

We included 7897 individuals aged on average 66 years from six intercontinental cohorts with blood metabolomic profiling (*n* = 1428 metabolites, of which 168 were present in at least three cohorts with over 80% prevalence) and MI information (1373 cases). We performed a two-stage individual patient data meta-analysis. We first assessed the associations between circulating metabolites and incident MI for each cohort adjusting for traditional risk factors and then performed a fixed effect inverse variance meta-analysis to pull the results together. Finally, we conducted a pathway enrichment analysis to identify potential pathways linked to MI. On meta-analysis, 56 metabolites including 21 lipids and 17 amino acids were associated with incident MI after adjusting for multiple testing (false discovery rate < 0.05), and 10 were novel. The largest increased risk was observed for the carbohydrate mannitol/sorbitol {hazard ratio [HR] [95% confidence interval (CI)] = 1.40 [1.26–1.56], *P* < 0.001}, whereas the largest decrease in risk was found for glutamine [HR (95% CI) = 0.74 (0.67–0.82), *P* < 0.001]. Moreover, the identified metabolites were significantly enriched (corrected *P* < 0.05) in pathways previously linked with cardiovascular diseases, including aminoacyl-tRNA biosynthesis.

**Conclusions:**

In the most comprehensive metabolomic study of incident MI to date, 10 novel metabolites were associated with MI. Metabolite profiles might help to identify high-risk individuals before disease onset. Further research is needed to fully understand the mechanisms of action and elaborate pathway findings.

## Graphical Abstract

**Graphical Abstract:**
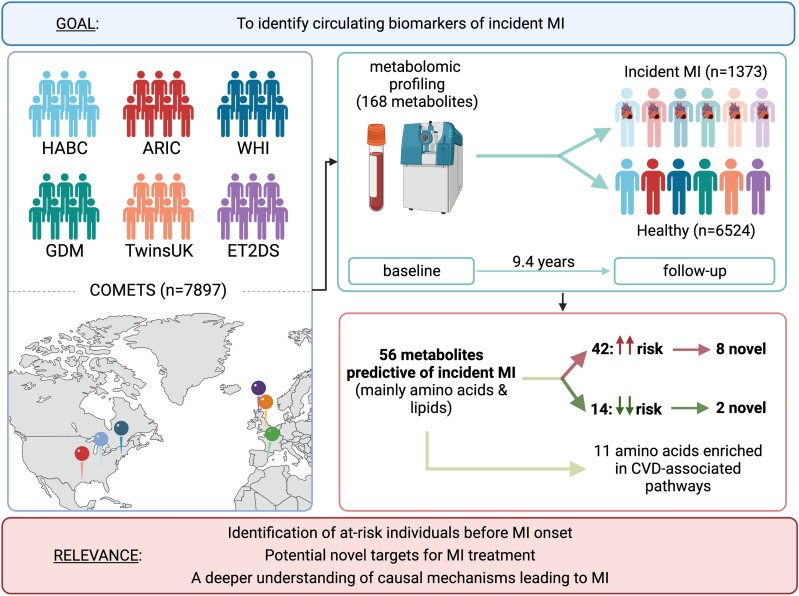

**Time of primary review: 48 days**

## 1. Introduction

Cardiovascular diseases (CVD) are a huge public health burden accounting for 32% of all global deaths in 2019.^1^ Myocardial infarction (MI) is one of the main causes of CVD, causing the death of one person every 40 s in the USA^2^ and one hospital admission every 5 min in the UK.^3^

Besides the well-established risk factors associated with MI, such as obesity, diabetes, hypertension, and smoking,^4^ many studies suggest that circulating metabolites might play an important role in MI development.^5,6^ For instance, glycine has been recognized as a protective biomarker of cardiac diseases, especially coronary heart disease,^7^ whereas trimethylamine *N*-oxide (TMAO) has been associated with MI by accelerating atherosclerosis.^5,6^

Metabolomics enables the comprehensive characterization of small-weight molecules, such as carbohydrates, amino acids, lipids, nucleotides, and peptides,^8–10^ providing a snapshot of the individual’s metabolic state at a particular time. Thus, metabolites might enable the identification of at-risk individuals before the disease process is well underway.^11,12^

Advances in this field have allowed the detection of metabolites whose deregulation may be involved in the onset and development of complex diseases including CVD,^13,14^ cancer,^15^ and autoimmune diseases.^16^ Nonetheless, most metabolomic studies are limited by the number of participants and/or the demographic diversity, affecting the statistical power of the results and hampering the discovery of potential universal biomarkers.^13,17^ To address these issues, the COnsortium of METabolomics Studies (COMETS) was established in 2014, aggregating metabolic data from 47 cohorts from around the world.^17^

By using individual patient data (IPD) from six COMETS cohorts with MI and metabolomic data, we aimed to identify biomarkers associated with incident MI in 7897 participants. We further explored the pathways in which these metabolites might be involved to better understand their mechanisms of action.

## 2. Methods

### 2.1 Study populations

**Figure 1:**
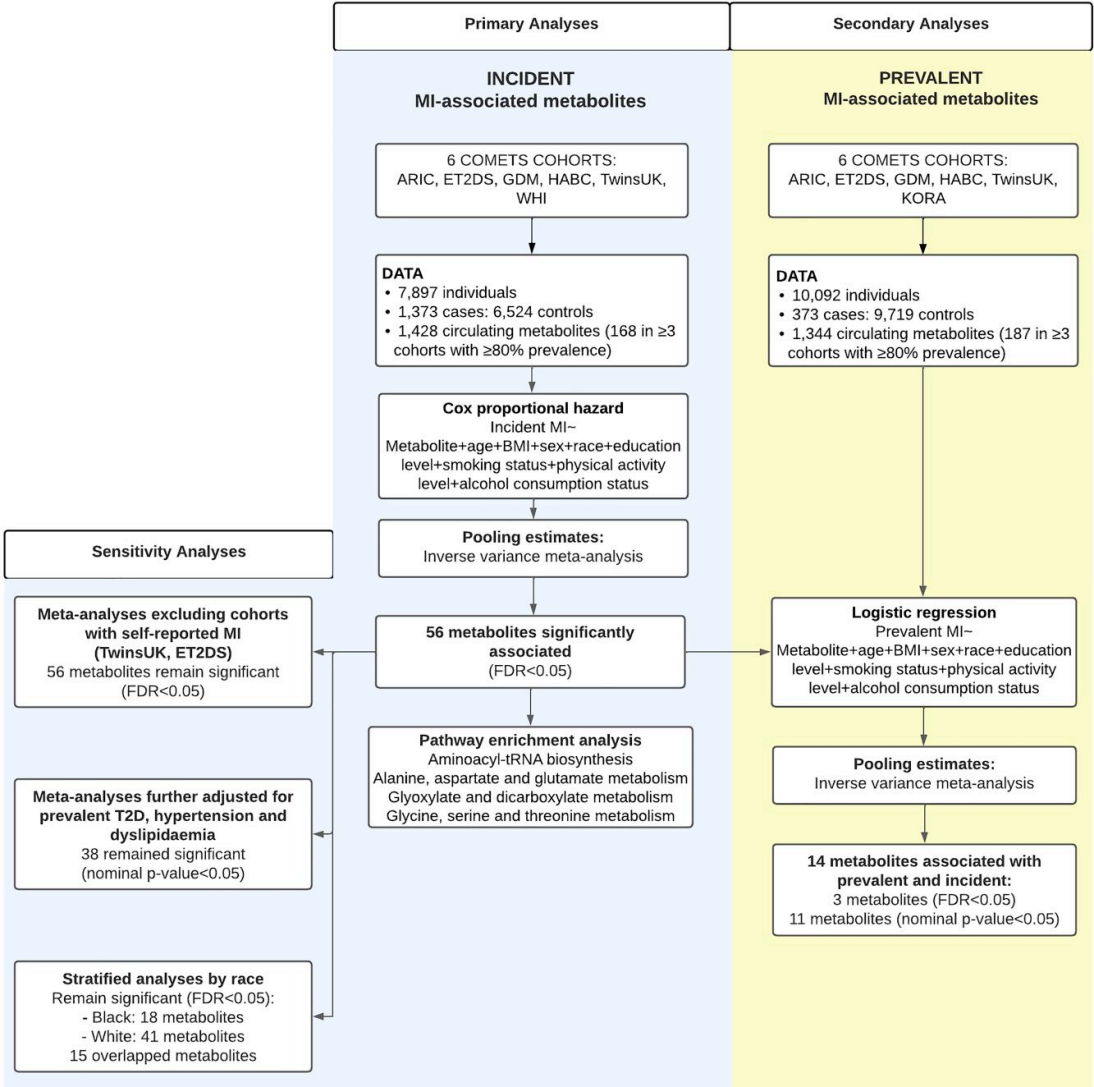
Flowchart overview containing the available data, steps conducted, and main results. ARIC, Atherosclerosis Risk in Communities; BMI, body mass index; ET2DS, Edinburgh Type 2 Diabetes Study; FRD, false discovery rate; GDM, GenoDiabMar; HABC, Health, Aging and Body Composition; KORA, Cooperative Health Research in the Region of Augsburg; WHI, Women’s Health Initiative.

For the primary analysis of metabolites associated with incident MI, we included participants from six population-based cohorts from the USA and Europe, namely, the Atherosclerosis Risk in Communities (ARIC) study, Edinburgh Type 2 Diabetes Study (ET2DS), GenoDiabMar (GDM), Health, Aging and Body Composition (HABC), TwinsUK, and the Women’s Health Initiative (WHI). Secondary analyses of metabolites associated with prevalent MI included participants from ARIC, ET2DS, GDM, HABC, TwinsUK, and Cooperative Health Research in the Region of Augsburg (KORA). Participants with available metabolomic data, covariates, and incident and prevalent MI data were included. Other COMETS cohorts could not be included in this study as they were lacking MI assessment and/or the metabolomic profile had not been performed by Metabolon Inc., the Broad Institute, or Nightingale Health. A flowchart of the study design is presented in *Figure 1*.

A brief description of the included COMETS cohorts is presented below and in *Table 1*.

- ARIC: Prospective cohort recruited from four US communities to investigate the aetiology of atherosclerosis and its clinical outcomes.^18^
- ET2DS: Longitudinal cohort of older men and women based in Lothian, Scotland, designed to investigate the role of risk factors for vascular complications of type 2 diabetes.^19^
- GDM: Prospective study that aims to provide data on demographic, biochemical, and clinical changes in type 2 diabetic patients attending real medical outpatient consultations.^20^
- HABC: Prospective cohort focused on risk factors for the decline of function in initially well-functioning older persons, particularly change in body composition with age.^21^
- KORA: A population-based adult cohort that consists of interviews, medical and laboratory examinations, biological sample collection, and multiple omic data generation and management.^25^
- TwinsUK: The largest most clinically characterized adult twin registry in the UK, recruited as volunteers without selecting for particular diseases or traits.^23^
- WHI: A large and complex clinical investigation of strategies for the prevention and control of some of the most common causes of morbidity and mortality among postmenopausal women, including cancer, CVD, and osteoporotic fractures.^13,24^

**Table 1:** Location and analytical information about the cohorts comprising COMETS

Cohort Name	Name abbreviation	Continent	Platform	Analytical technology	Targeted/untargeted	Description
Atherosclerosis Risk in Communities Study	ARIC	North America	Metabolon	GC/LC-MS	Untargeted	Prospective cohort recruited from four US communities to investigate the aetiology of atherosclerosis and its clinical outcomes^18^
Edinburgh Type 2 Diabetes Study	ET2DS	Europe	Nightingale	NMR	Targeted	Longitudinal cohort of older men and women based in Lothian, Scotland, designed to investigate the role of risk factors for vascular complications of type 2 diabetes^19^
GenoDiabMar	GDM	Europe	Nightingale	NMR	Targeted	Prospective study that aims to provide data on demographic, biochemical, and clinical changes in type 2 diabetic patients attending real medical outpatient consultations^20^
Health, Aging and Body Composition	HABC	North America	Broad Institute	LC-MS	Untargeted	Interdisciplinary cohort focused on risk factors for the decline of function in initially well-functioning older persons, particularly change in body composition with age^21^
Cooperative Health Research in the Region of Augsburg	KORA	Europe	Metabolon	GC/LC-MS	Untargeted	A population-based adult cohort and initiated as part of the World Health Organization Multinational Monitoring of Trends and Determinants in Cardiovascular Diseases (MONICA) project since 1984^22^
TwinsUK	TwinsUK	Europe	Metabolon	GC/LC-MS	Untargeted	The largest most clinically characterized adult twin registry in the UK, recruited as volunteers without selecting for particular diseases or traits^23,25^
Women’s Health Initiative	WHI	North America	Broad Institute	LC-MS	Untargeted	A large and complex clinical investigation of strategies for the prevention of some of the most common causes of morbidity and mortality among postmenopausal women, including cancer, cardiovascular disease, and osteoporotic fractures.^13,24^

### 2.2 Metabolomics

A summary of the metabolomics methodology used for each cohort is depicted in *Table 1*. Serum samples from ARIC, ET2DS, GDM, KORA, and TwinsUK and samples of ethylenediaminetetraacetic acid (EDTA) plasma from HABC, TwinsUK, and WHI were held at −80°C.^17^ Serum metabolites were detected and quantified in ARIC, KORA, and TwinsUK at Metabolon Inc. using untargeted gas chromatography/liquid chromatography-mass spectrometry (GC/LC-MS) methods, in ET2DS and GDM at Nightingale Health using a nuclear magnetic resonance (NMR) method. EDTA plasma metabolites were detected and quantified in HABC and WHI at the Broad Institute using LC-MS. Metabolites were harmonized across platforms by manual curation by matching chemical structure, and the Human Metabolon Database and Kyoto Encyclopedia of Genes and Genomes (KEGG) identifiers. A total of 1442 unique named and known metabolites were measured across seven participating studies. For the primary analysis, we included 1428 metabolites, from which 168 were present in at least three studies and detected in at least 80% of participants from each cohort. For the secondary analysis, measurements of 1344 metabolites were available (from which 187 were present in at least three studies and detected in at least 80% of participants from each cohort). In this study, our focus is to explore the metabolites significantly associated with incident MI and the pathways in which are enriched. The prevalent analysis aimed to explore the overlap of metabolites associated with incident and prevalent MI.

### 2.3 Assessment of MI and co-variables

Specific information about how each cohort defined MI is shown in Supplementary material online, *Text S1*. In summary, MI was assessed based on one or more of the following:

- Diagnosed by a doctor (based on clinical evidence such as chest pain, electrocardiogram, and cardiac enzymes).
- Self-reported questionnaires.
- Hospital/GP records.
- Death certificates including the adjudication.

On the other hand, co-variables used to adjust the models were described identically across the cohorts. How these were defined is indicated in Supplementary material online, *Text S1*.

### 2.4 Statistical analysis

We conducted a two-step IPD meta-analysis. In the first step, we performed analyses separately by study cohort. Outliers defined as values four standard deviations (SDs) from the mean were excluded. To obtain normal distributions, metabolite measures were transformed to rankits by performing quantile normalization on rank-transformed raw metabolite values. Power calculation was performed using the ‘dmetar’ package implemented in R. For each metabolite included in the primary analysis, Cox proportional hazard models for incident MI were fit adjusting for age, sex, race/ethnicity, body mass index (BMI), education level, smoking status, physical activity level, and alcohol consumption status, all at the baseline visit. In the second step, we meta-analysed the results from each cohort using fixed effect inverse variance meta-analyses (using the package ‘meta’ in R) for metabolites present in three or more studies. Heterogeneity between studies and percentage of variability of between-study heterogeneity not due to the sampling error were computed using Cochran’s Q test and *I*^2^ index, respectively.

Sensitivity analyses were conducted by (i) running Han–Eskin random effect meta-analyses^26^; (ii) further adjusting for prevalent type 2 diabetes, prevalent hypertension, and prevalent dyslipidaemia; (iii) excluding cohorts where MI was assessed through self-reported questionnaires (e.g. TwinsUK and ET2DS); and (iv) stratifying by race (White individuals and Black individuals).

Secondary analyses were conducted to assess the associations between metabolites and prevalent MI using two-step IPD meta-analysis. Logistic regression models were first run in each cohort on rankit transformed metabolite measures adjusting for the same covariates, and then a fixed effect inverse variance meta-analysis was performed.

We adjusted for multiple testing using Benjamini and Hochberg^27^ false discovery rate (FDR <0.05). If not indicated otherwise, all reported *P*-values are FDR-adjusted. Analyses were undertaken and reported according to the STrengthening the Reporting of OBservational studies in Epidemiology (STROBE) guidelines (see Supplementary material online, *Text S2*). We define that a metabolite is novel when, to our knowledge, such a metabolite has never been associated with any cardiac disease before.

### 2.5 Metabolomic pathway analysis

To explore the metabolomic pathways enriched for MI-related metabolites, we used MetaboAnalyst 5.0.^28^ Over-representation analysis was performed using a hyper-geometric test to identify groups of compounds that are represented more than expected in each pathway by chance, and pathway topology analysis was performed based on relative betweenness centrality focusing on our entire metabolomic network. Metabolites significantly associated with incident MI (FDR < 0.05) were mapped to the *Homo sapiens* KEGG pathways. Metabolomic pathways with FDR < 0.05 were considered statistically significant.

### 2.6 Ethical approval

Approval was granted by the COMETS steering committee. Ethical approval for each study was obtained by the ethical research boards pertaining to each study.

## 3. Results

The descriptive characteristics of the study participants are shown in *Table 2*. We included 7897 individuals [average age = 66 years (SD = 7.1)] with blood metabolomic profiling (*n* = 1428 metabolites) and incident MI assessment from six cohorts including ARIC, ET2DS, GDM, HABC, TwinsUK, and WHI. All included participants were free from MI at baseline. There were 1373 incident MI cases across the six cohorts [average follow-up time = 9.4 years (SD = 7.1); average follow-up time per cohort is presented in *Table 2*]. For the secondary analysis, we included 373 prevalent MI cases and 9719 prevalent MI controls from the ARIC, ET2DS, GDM, HABC, TwinsUK, and KORA cohorts (descriptive characteristics are shown in *Table 2*).

**Table 2:** Descriptive characteristics at baseline of the participants from the COMETS cohorts containing incident and/or prevalent myocardial infarction data

Cohort (Metabolite number)	MI type	Subsets	Sample Size, *N*	Women, %	Baseline Age, years	Follow-up Age, years	BMI, kg/m^2^	Race, %	Follow-up Time, years
ARIC (*n* = 311)	Incident	All participants	3776	61	53 (5.7)	76 (8.7)	28.8 (5.9)	38% White, 62% Black	22.8 (8.4)
		MI cases	442	55	55 (5.7)	70 (9)	29.3 (5.4)	41% White, 59% Black	15.5 (8)
		Controls	3334	62	53 (5.7)	77 (8.3)	28.7 (5.9)	38% White, 62% Black	23.8 (7.8)
	Prevalent	All participants	3395	62	53 (5.8)	–	28.7 (5.9)	38% White, 62% Black	–
		MI cases	54	33	57 (5.7)	–	29.1 (5.1)	56% White, 44% Black	–
		Controls	3341	62	53 (5.7)	–	28.7 (5.9)	38% White, 62% Black	–
ET2DS (*n* = 208)	Incident	All participants	909	53	68 (4.2)	77 (4.6)	31.4 (5.8)	98% White, 2% non-White^b^	9.5 (2.8)
		MI cases	66	47	69 (3.8)	75 (4.9)	31.2 (5.4)	98% White, 2% non-White^b^	5.9 (3.1)
		Controls	843	53	68 (4.2)	77 (4.6)	31.4 (5.8)	98% White, 2% non-White^b^	9.8 (2.6)
	Prevalent	All participants	992	49	68 (4.2)	–	31.4 (5.7)	98% White, 2% non-White^b^	–
		MI Cases	147	22	69 (4.1)	–	31.3 (5.2)	98% White, 2% non-White^b^	–
		Controls	845	53	68 (4.2)	–	31.5 (5.8)	98% White, 2% non-White^b^	–
GDM (*n* = 210)	Incident	All participants	477	41	69 (9.3)	73 (9.1)	30.3 (5.2)	100% White	4.4 (1.3)
		MI cases	42	33	70 (8.4)	73 (8.2)	30.1 (5.3)	100% White	2.3 (1.6)
		Controls	435	42	69 (9.4)	74 (9.2)	30.4 (5.2)	100% White	4.5 (1.2)
	Prevalent	All participants	468	41	69 (9.4)	–	30.4 (5.2)	100% White	–
		MI cases	33	33	71 (10.1)	–	31.5 (4.8)	100% White	–
		Controls	435	42	69 (9.4)	–	30.4 (5.2)	100% White	–
HABC (*n* = 350)	Incident	All participants	236	0	75 (2.8)	83 (4.7)	27.0 (4.5)	100% Black	10.6 (5)
		MI cases	25	0	75 (2.8)	83 (4.7)	27.0 (4.6)	100% Black	7.6 (3.7)
		Controls	211	0	75 (2.9)	81 (4.4)	26.8 (3.2)	100% Black	10.6 (5.1)
	Prevalent	All participants	1764	0	75 (2.8)	–	27.0 (4.5)	100% Black	–
		MI cases	63	0	75 (2.8)	–	27.6 (4.6)	100% Black	–
		Controls	1701	0	74 (2.8)	–	26.8 (4.4)	100% Black	–
TwinsUK (*n* = 591)	Incident	All participants	911	97	65 (8)	70 (7.7)	26.1 (4.8)	100% White	3.9 (2.9)
		MI cases	5	80	74 (5.2)	77 (5.2)	31.7 (9.6)	100% White	2.6 (0.1)
		Controls	906	97	66 (8)	70 (7.7)	26.1 (4.7)	100% White	3.9 (2.9)
	Prevalent	All participants	1708	97	65 (8.6)	–	26.3 (4.8)	100% White	–
		MI cases	13	77	71 (5.8)	–	28.5 (5.8)	100% White	–
		Controls	1695	97	65 (8.6)	–	26.3 (4.8)	100% White	–
WHI (*n* = 414)	Incident	All Participants	1588	100	67 (6.9)	72 (7.5)	28.4 (6.1)	77% White, 23% non-White^a^	5.1 (3.3)
		MI cases	793	100	67 (7.0)	72 (7.5)	29.0 (6.3)	77% White, 23% non-White^a^	5.1 (3.3)
		Controls	795	100	67 (6.9)	72 (7.4)	27.9 (5.9)	77% White, 23% non-White^a^	5.1 (3.3)
KORA (*n* = 353)	Prevalent	All participants	1765	52	61 (8.8)	–	28.2 (4.8)	100% White	–
		MI cases	63	22	67 (6.6)	–	30.7 (5.1)	100% White	–
		Controls	1702	53	61 (8.8)	–	28.1 (4.8)	100% White	–
Total (unique: *n* = 1428)	Incident	All participants	7897	59	66 (7.1)	75 (4.6)	28.7 (2)	69% White, 29% Black, 2% Others	9.4 (7.1)
		MI cases	1373	53	68 (7.3)	75 (4.6)	29.7 (1.7)	69% White, 29% Black, 2% Others	6.5 (4.8)
		Controls	6524	59	66 (7.2)	75 (4)	28.6 (2.1)	69% White, 29% Black, 2% Others	9.6 (7.5)
	Prevalent	All participants	10 092	50	65 (7.4)	–	28.7 (2)	73% White, 27% Black^c^	–
		MI cases	373	31	68 (6)	–	29.8 (1.6)	76% White, 24% Black^c^	–
		Controls	9719	51	65 (7.2)	–	28.6 (2)	73% White, 27% Black^c^	–

^a^In the WHI, non-White included: 14% Black or African-American, 3% Hispanic/Latino, 2% Asian or Pacific Islander, and 4% others.

^b^In the ET2DS, non-White included for prevalent: 1.1% Asian, 0.2% Black, 0.1% White and Black Caribbean, and 0.1% White and Asian. Specifically, 98.4% are White. Non-White included for incident: 1.1% Asian, 0.2% Black, 0.1% White and Black Caribbean, and 0.1% White and Asian. Specifically, 98.5% are White.

^c^It presents <1% of other ethnicities (non-White and non-Black).

### 3.1 Metabolites associated with incident MI

**Figure 2:**
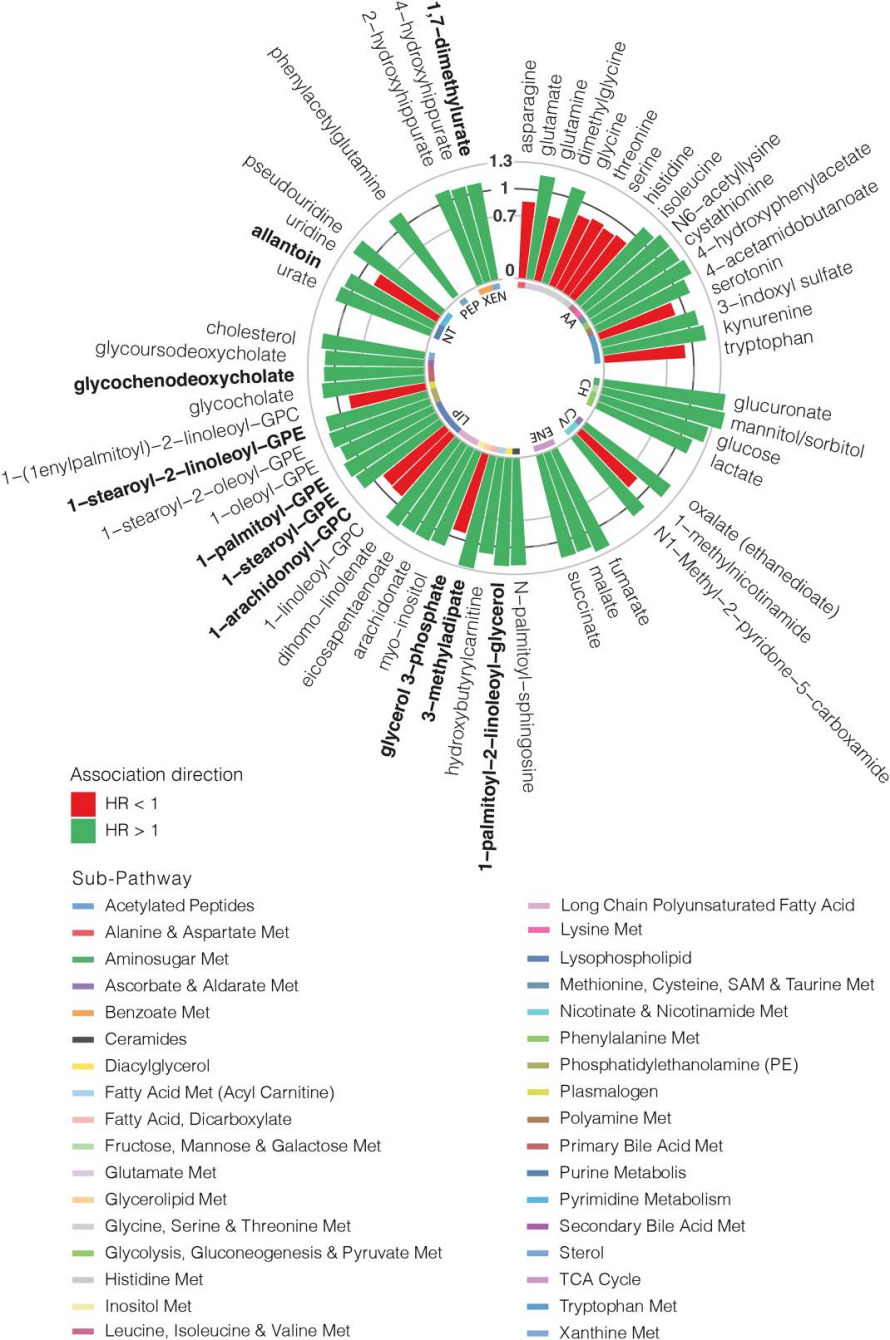
Metabolites significantly associated with incident myocardial infarction. The bar height represents the hazard ratio (HR) value. Novel metabolites are highlighted in bold. Each metabolite super-pathway and sub-pathway is also indicated. AA, amino acid; CH, carbohydrate; C/V, co-factors/vitamins; ENE, energy; LIP, lipid; Met, metabolite; NT, nucleotide; XEN, xenobiotic.

For our primary analysis including 1373 incident MI cases and 6524 controls, assuming a modest effect size of 0.12 [corresponding to hazard ratio (HR) = 1.127 or HR = 0.887], our study has over 90% power for a given metabolite adjusting for multiple testing (*P* < 3.5 ∗ 10^−5^). We meta-analysed 1428 metabolites, of which 168 were present in at least 80% of the participants from at least three studies. In total, 56 metabolites were significantly associated with incident MI after adjusting for multiple testing (FDR < 0.05) (*Figure 1*; see Supplementary material online, *Table S1*). Out of the 56 metabolites, 42 had a direct association, and 14 had an inverse association with incident MI (*Figure 2*). Moreover, 21 were lipids, primarily lysophospholipids (*n* = 5), long-chain polyunsaturated fatty acids (*n* = 3), phosphatidylethanolamine (*n* = 2), and products of the primary bile acid metabolism (*n* = 2), and 17 were amino acids including products of tryptophan metabolism (*n* = 4), glycine, serine, and threonine (*n* = 4) and glutamate metabolism (*n* = 2). There were also 4 nucleotides, 4 carbohydrates, 3 xenobiotics, 3 energy-producing metabolites, 3 co-factors/vitamins, and 1 peptide (*Figure 2*). Out of the 21 associated lipids, 3-methyladipate and 1-palmitoyl-2-linoleoyl-glycerol (16:0/18:2) were associated with a higher risk with HR estimates ranging from 1.28 [95% confidence interval (CI) = 1.13–1.44, *P* < 0.001] to 1.21 (95% CI = 1.08–1.35, *P* = 4.29 × 10^−3^), respectively (*Figure 2*). Among the amino acids, 4-hydroxyphenylacetate and cystathionine had the largest increase in risk presenting HR estimates of 1.24 (95% CI = 1.11–1.38, *P* = 1.11 × 10^−3^) and 1.2 (95% CI = 1.07–1.35, *P* = 7.58 × 10^−3^), respectively (*Figure 2*). Likewise, overall, the highest increase of risk was observed for the carbohydrates mannitol/sorbitol [HR (95% CI) = 1.40 (1.26–1.56), *P* < 0.001] and glucuronate [HR (95% CI) = 1.37 (1.26–1.5), *P* < 0.001], whereas the metabolites associated with reduced risk of incident MI included the amino acid glutamine [HR (95% CI) = 0.74 (0.67–0.82), *P* < 0.001], the nucleotide uridine [HR (95% CI) = 0.82 (0.76–0.88), *P* < 0.001], and the co-factor 1-methylnicotinamide [HR (95% CI) = 0.84 (0.76–0.94), *P* = 7.37 × 10^−3^], among others (*Figure 2*). The list of metabolites previously associated with any cardiac diseases and the super- and sub-pathways for incident MI-associated metabolites are presented in Supplementary material online, *Table S2*.

Of note, the obtained heterogeneity estimated for the associated metabolites was only significant (Q *P* < 0.05) for seven metabolites with also *I*^2^ values indicating considerable variability of between-study heterogeneity (*I*^2^ > 70%).^29^ However, most identified metabolites presented not relevant or moderate between-study heterogeneity (*I*^2^ < 60%).^29^

### 3.2 Sensitivity analyses

Results were consistent when running Han–Eskin random effect inverse variance meta-analyses^26^ (see Supplementary material online, *Table S3*). Results were also consistent when the meta-analysis was performed excluding cohorts in which MI was assessed by self-reported questionnaires (i.e. TwinsUK and ET2DS) (see Supplementary material online, *Table S4*). When we further adjusted for prevalent type 2 diabetes, hypertension, and dyslipidaemia, 38 metabolites remained associated (see Supplementary material online, *Table S5*). Interestingly, the metabolites that did not reach the significance level after adjustment for co-morbidities have been previously linked with those commodities (see Supplementary material online, *Table S2*). Finally, we investigated whether there were demographic differences in the associations between the identified metabolites and MI by conducting a meta-analysis stratified by race. Out of the 56 metabolites, 41 remained significantly associated in White individuals, whereas 18 were significantly associated in Black individuals, with 3 of them, namely, dimethylglycine, glycine, and glycoursodeoxycholate, presenting a significant association only in individuals with an African ancestry (see Supplementary material online, *Table S6*).

### 3.3 Metabolites associated with prevalent MI

As a secondary analysis, we further investigated whether the 56 metabolites associated with incident MI were also correlated with prevalent MI (*Figure 1*). On meta-analyses, 11 metabolites, including tryptophan, malate, allantoin, and 1-linoleoyl-GPC (18:2), were nominally associated with prevalent MI with concordant directional effects in both incident and prevalent analyses, and three [xenobiotic 2-hydroxyhippurate (salicylurate), lactate, and glucoronate] were associated after correcting for multiple testing [2-hydroxyhippurate: odds ratio (OR) (95% CI) = 1.9 (1.5–2.42), *P* < 0.001; lactate: OR (95% CI) = 1.36 (1.2–1.54), *P* < 0.001; and glucuronate: OR (95% CI) = 1.51 (1.19–1.93), *P* = 0.03] (see Supplementary material online, *Table S7*).

### 3.4 Pathways behind the metabolites associated with incident MI

**Figure 3:**
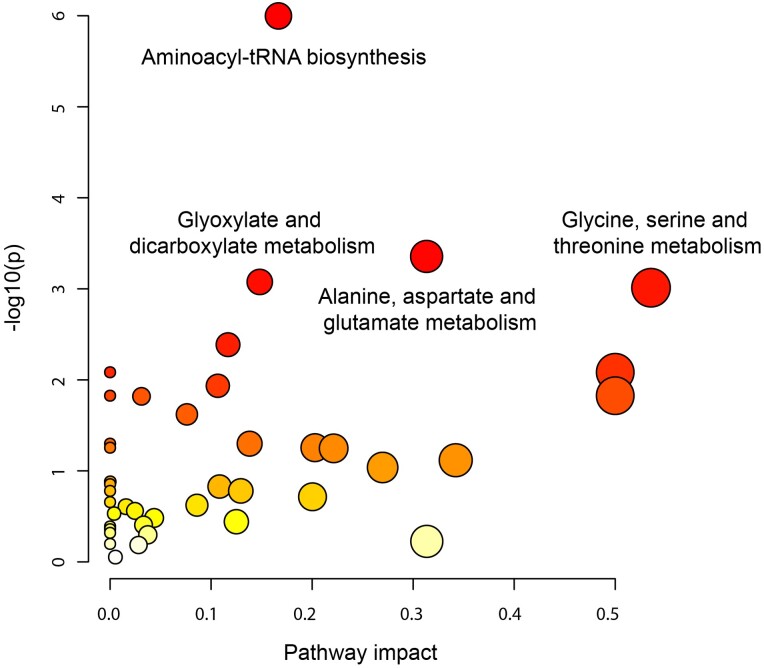
Enrichment pathway analysis results indicating the significant pathways (FDR < 0.05) among the identified metabolites associated with incident myocardial infarction.

To identify the potential biological pathways involved in incident MI, we assessed the enriched pathways for the 56 metabolites (*Figure 1*). These metabolites included 41 pathways, 12 of which had a significant nominal *P*-value, including the citrate cycle [trichloroacetic acid (TCA) cycle] (nominal *P* = 0.016) and the primary bile acid biosynthesis (nominal *P* = 0.024) (see Supplementary material online, *Table S8*). Of these 12, 4 pathways were significantly enriched (FDR < 0.05), namely, aminoacyl-tRNA biosynthesis (*P* < 0.001), alanine, aspartate, and glutamate metabolism (*P* = 0.018), glyoxylate and dicarboxylate metabolism (*P* = 0.02), and glycine, serine, and threonine metabolism (*P* = 0.02) (*Figure 3*). Specifically, 9 amino acids were involved in the 1st pathway, 3 amino acids and the energy-producing metabolites fumarate and succinate in the 2nd pathway, 4 amino acids and the energy-producing metabolite malate in the 3rd pathway, and 5 amino acids in the 4th pathway (see Supplementary material online, *Table S8*). There were 14 unique metabolites involved in these four pathways. Glycine and serine are intermediates/products of aminoacyl-tRNA biosynthesis; glycine, serine, and threonine metabolism; and glyoxylate and dicarboxylate metabolism, whereas glutamine and glutamate are present in all the pathways but the glycine, serine, and threonine metabolism.

## 4. Discussion

In this comprehensive study investigating biomarkers of incident MI by leveraging IPD from six intercontinental cohorts with 7897 participants from diverse race/ethnic backgrounds, we identified 56 metabolites, mainly lipids and amino acids, significantly associated with incident MI. We report 10 novel biomarkers of incident MI, including 8 lipids (3 lysophospholipids, 1 phosphatidylethanolamine, 1 diacylglycerol, 1 intermediate of the primary bile acid metabolism, 1 dicarboxylate fatty acid, and 1 glycerolipid), 1 xenobiotic (involved in xanthine metabolism), and 1 nucleotide (involved in purine metabolism). Of these, 6 have underlying mechanisms of action leading to MI onset which are independent of hypertension, type 2 diabetes, and dyslipidaemia, known as risk factors for MI.^14,30–32^ We also confirm previous associations, including the protective association of nonessential amino acids (e.g. glutamine, glycine, and serine),^7,33^ and the detrimental effect of the well-known branched-chain amino acid isoleucine on cardiac diseases,^34^ thus demonstrating the robustness of our approach. Our stratified analyses revealed that dimethylglycine, glycine, and glycoursodeoxycholate were associated with incident MI only in Black individuals, highlighting the role of ethnicity in the aetiology of MI. We also show that the metabolites that might lead to the MI onset differ from the metabolites deregulated once the disease is well established, highlighting the importance of survival analyses to identify preventive biomarkers. Finally, we report the pathways in which the identified amino acids are enriched, shedding light on the mechanisms by which these metabolites may be implicated in MI onset. Of note, most of the identified metabolites are lipids, and enrichment of lipid metabolism pathways was observed, but these did not attain statistical significance due to the involvement of many metabolites and thus the need for a large overlap with the lipid-associated MI to be considered significant. This complexity underscores the intricate nature of lipid metabolism pathways, and the multiple roles lipids play in the onset of MI.

### 4.1 Lysophospholipids

Among the lipids, lysophospholipids represent the largest subgroup found to be associated with incident MI. Specifically, we identified 5 metabolites belonging to this sub-pathway, with 3 of them, namely, 1-oleoyl-GPE (18:1), 1-palmitoyl-GPE (16:0), and 1-stearoyl-GPE (18:0), associated with an increased risk of MI and two of them, 1-linoleoyl-GPC (18:2) and 1-arachidonoyl-GPC (20:4), associated with decreased risk of MI. Of these, 1-palmitoyl-GPE (16:0), 1-arachidonoyl-GPC (20:4), and 1-stearoyl-GPE (18:0) are novel biomarkers of MI. Lysophospholipids are a group of bioactive molecules with diverse biological roles, including activation of specific G-protein-coupled receptors, and have been associated with atherosclerosis, coronary heart disease, and hypertension.^35^ Nonetheless, their effects on CVD are controversial as both beneficial and detrimental effects have been reported. For instance, they might possess cardioprotective effects, but, also, they might stimulate platelet aggression, enhancing ischaemia in MI.^35^ This fact along with the opposing results found between these metabolites and MI might indicate that lysophospholipids’ function might vary depending on their subclasses.

### 4.2 Intermediates of bile acid metabolism

Here, we report for the first time that incident MI cases have higher circulating levels of the secondary bile acid glycochenodeoxycholate compared to controls. Bile acids can act as signalling molecules involved in inflammatory processes and host metabolism.^36^ Several CVD metabolomics studies have highlighted the negative role of bile acids on CVD morbidity/mortality.^37,38^ Glycochenodeoxycholate is a bile acid-lycine conjugate produced by the gut microbiota.^39^ Studies have reported glycochenodeoxycholate is toxic and can induce hepatocyte apoptosis, which might lead to liver disease.^40^ Likewise, liver and cardiac diseases co-exist through complex cardio hepatic interactions.^41^ Our results may suggest that high levels of this bile acid can have detrimental effects on MI by causing alterations in the liver, and the gut microbiota might be targeted to modulate its levels.

### 4.3 Nucleotide metabolism intermediates

We are the first to report the association between allantoin and MI. Allantoin is involved in purine metabolism and is formed from the oxidation of urate by various reactive oxygen species.^42^ Allantoin has been reported as a potential marker of oxidative stress in humans,^42^ possibly explaining the observed positive association with MI. Moreover, we show the associations of pseudouridine and uridine, intermediates of the pyrimidine metabolism, and also urate, involved in the purine metabolism, with incident MI. This confirms previous findings and points out the important role of the nucleotide metabolism intermediates in cardiovascular risk.^38^ For instance, hyperuricaemia has been shown to be strongly positively associated with carotid and coronary vascular disease and stroke.^43^

### 4.4 Co-factors involved in the nicotinate and nicotinamide metabolism

We identified 3 co-factors associated with incident MI, from which 1-methylnicotinamide and N1-methyl-2-pyridone-5-carboxamide were intermediates of the nicotinate and nicotinamide metabolism. 1-Methylnicotinamide presented an important protective effect in MI, which is concordant with their shown antithrombotic action in rats.^44^ On the contrary, N1-methyl-2-pyridone-5-carboxamide was negatively associated with MI, and to our knowledge, no studies have previously reported such an association with incident MI. Nonetheless, Surendran and colleagues^45^ stated changes in its plasma levels during myocardial ischaemia-reperfusion injury. N1-Methyl-2-pyridone-5-carboxamide has been reported as a uremic toxin.^46^ These are organic compounds that accumulate in the bloodstream, as they cannot be eliminated from the body, reaching diverse organs, including the heart,^47^ and they are a risk factor for the progression of chronic kidney disease. Likewise, patients with chronic kidney disease have an increased risk for CVD, for instance, these molecules can lead to vascular damage by enhancing the expression of cytokines and pro-inflammatory molecules.^47^

### 4.5 Amino acids

Pathway enrichment analysis revealed that 11 incident MI-associated amino acids are enriched in pathways previously associated with CVD. Firstly, the aminoacyl-tRNA biosynthesis pathway has been reported to be closely related to angiogenesis and cardiomyopathy.^48^ Likewise, the glyoxylate and dicarboxylate metabolism is another commonly disturbed pathway found in different CVD.^49^ Eventually, the metabolism of glycine, serine, and threonine has been linked with benefits in atherosclerosis,^50^ being concordant with the found negative associations of glycine, serine, and threonine with incident MI. Of note, these pathways share most of the included metabolites and are characterized for being sensitive to the amino acids availability,^48^ suggesting that deregulation of the matched amino acids might lead to different cardiovascular complications, including MI, and emphasizes the importance of a balanced amino acid profile.

Our study has some limitations. Firstly, the number of healthy participants is 5.7-fold larger than the number of incident MI cases, although we have been able to identify 56 metabolites whose levels significantly differ between MI cases and controls. Secondly, the clinical definition of MI varies in each cohort depending on the protocol for data collection. This may introduce a procedural bias. However, when we ran a sensitivity analysis by excluding cohorts where MI was assessed by self-reported questionnaires, the results remained consistent. Thirdly, metabolomics profiling was conducted using different metabolomic platforms, raising some caveats: (i) a different, somehow overlapping, set of metabolites was measured by each platform, and we are only including metabolites present in at least three cohorts; (ii) we quantile normalized metabolites to meta-analyse results across studies using different metabolomic platforms. However, ranks do not have practical significance and could be influenced by the sample size; (iii) metabolite sampling and detection times could not be unified as each cohort applies used a different metabolomics methodology. Fourth, though metabolite concentrations might be influenced by medications (e.g. statins),^51^ we were unable to adjust for drug usage as the data were not available across the studies. Statins are the main therapy for the worldwide prevention of CVD, including MI.^52,53^ They inhibit the rate-limiting step in cholesterol synthesis, thereby lowering serum cholesterol levels and reducing MI risk.^54^ Statins can also reduce MI risk via cholesterol-independent mechanisms, for instance, by inhibiting the isoprenoid synthesis.^55^ Hence, statin usage and adherence could be confounding our results, and this should be addressed in future studies. Fifth, our study sample was predominantly White, and some MI-associated metabolites might have not reached the significance level in Black individuals due to lack of power. Future studies should further investigate race–metabolite interactions^56^ to better understand the role of race in the metabolite–MI association. Finally, it is important to note that these results do not necessarily imply causality.

Notwithstanding the above limitations, our study benefits from a two-step meta-analysis using IPD, which has been recognized as a ‘gold standard’ to evidence synthesis,^57^ and a high number of participants, which increases the power of our statistical analyses and minimizes the chances of obtaining false positives. Also, sensitivity analyses were run stratifying by race, allowing us to investigate the influence of demographic diversity in the identified associations. Furthermore, measurements of a wide range of metabolites, belonging to different pathways and sub-pathways, were available for each cohort allowing us to obtain a wide picture of the role played by metabolomics in MI. Different platforms were used for the metabolite measurements, reducing the inclusion of measurement errors or misidentified metabolites given by a certain platform. Moreover, despite using distinct platforms and manners to define MI, the significance of the identified metabolites was concordant across cohorts. Finally, the prospective nature of the current study permitted us to investigate how distinct metabolomic profiles are associated with incident MI.

In conclusion, these findings shed light on novel metabolic preventive biomarkers of MI and the involved pathways and might help to identify high-risk individuals before the disease onset and pave the way towards the development of novel preventative strategies. Nonetheless, more research needs to be conducted to confirm the identified metabolites as biomarkers and to fully understand underlying the mechanisms of action.

## Supplementary material

Supplementary material is available at *Cardiovascular Research* online.

## Supplementary Material

cvad147_Supplementary_Data

## Data Availability

The phenotypic data used from the Atherosclerosis Risk in Communities (ARIC) Cohort are assessed via dbGaP (Study Accession: phs000280.v8.p2) or BioLINCC (https://biolincc.nhlbi.nih.gov/studies/aric/). The ARIC metabolomic data can be requested through the study's Data Coordinating Center upon an approved manuscript proposal and Data and Materials Distribution Agreement (DMDA). ET2DS can only share with bonafide researchers under managed access and when local resources are available for historical data management. GDM data available upon reasonable request from the author CB due to patient's privacy/ethical restrictions. HABC can only share with approved investigators under managed access. The KORA FF4 datasets are available upon application through the KORA-PASST (Project application self-service tool, https://www.helmholtz-munich.de/epi/research/cohorts/kora-cohort/data-use-and-access-viakorapasst/index.html.) The TwinsUK data are held by the Department of Twin Research at King's College London. The data can be released to bona fide researchers using our normal procedures overseen by the Wellcome Trust and its guidelines as part of our core funding (https://twinsuk.ac.uk/resources-for-researchers/access-our-data/). WHI data is publicly available in DbGAP.

## Notes

Translational perspective In the largest meta-analyses covering six international cohorts, we identify 10 novel and 46 known metabolites associated with incident MI that can be used to identify at-risk individuals before disease onset. Our results improve our understanding of the molecular changes that take place in MI development and provide potential novel targets for clinical prediction and a deeper understanding of causal mechanisms.
