# Supplementary material for: Predictive metabolites for incident myocardial infarction: a two-step meta-analysis of individual patient data from six cohorts comprising 7897 individuals from the COnsortium of METabolomics Studies
Source: Cardiovasc Res. 2023 Sep 14;119(17):2743–54. doi: 10.1093/cvr/cvad147 (PMC10757581; doi:10.1093/cvr/cvad147)
Supplement: cvad147_Supplementary_Data [file cvad147_supplementary_data.zip › MI_COMETS supplementary legends.docx]

Predictive metabolites for incident myocardial infarction: a two-step meta-analysis of individual patient data from six cohorts comprising 7,897 individuals from the the COnsortium of METabolomic Studies

Ana Nogal^1*^, Taryn Alkis^2*^, Yura Lee^2*^, Domagoj Kifer^3^, Jie Hu^4^, Rachel A. Murphy^5,6^, Zhe Huang^7^, Rui Wang-Sattler^8^, Gabi Kastenmüler^9^, Birgit Linkohr^10^, Clara Barrios^11^, Marta Crespo^11^, Christian Gieger^8^, Annette Peters^10^, Jackie Price^7^, Kathryn M. Rexrode^4^, Bing Yu^2§^, Cristina Menni^1§^

**Author’s affiliations:**

^1^ Department of Twin Research, King’s College London, St Thomas’ Hospital Campus, London SE1 7EH.

^2^ Department of Epidemiology, Human Genetics and Environmental Sciences, University of Texas Health Science Center at Houston School of Public Health, Houston.

^3^ Faculty of Pharmacy and Biochemistry, University of Zagreb, Zagreb, Croatia.

^4^ Division of Women’s Health, Department of Medicine, Brigham and Women’s Hospital, Boston, Massachusetts.

^5^ Faculty of Medicine, University of British Columbia, Vancouver, BC.

^6^ Cancer Control Research, BC Cancer, Vancouver BC

^7^ Usher Institute of Population Health Sciences and Informatics, University of Edinburgh, Edinburgh, Scotland, United Kingdom.

^8^ Research Unit of Molecular Epidemiology, Helmholtz Zentrum München, Neuherberg, Germany.

^9^ Institute of Bioinformatics and Systems Biology, Helmholtz Zentrum München, Neuherberg, Germany.

^10^ Institute of Epidemiology, Helmholtz Zentrum München, Neuherberg, Germany.

^11^ Department of Nephrology, Hospital del Mar, Institut Hospital del Mar d´Investigacions Mediques, Barcelona, Spain.

*Equal contribution

^§^Equal contribution

**Corresponding authors:**

Cristina Menni, PhD

Department of Twin Research, King’s College London, St Thomas’ Hospital Campus, Westminster Bridge Road, London SE1 7EH, UK

Phone: +44 (0) 207 188 7188 (ext. 52594); email: [cristina.menni@kcl.ac.uk](mailto:cristina.menni@kcl.ac.uk)

Bing Yu, PhD

Department of Epidemiology, Human Genetics and Environmental Sciences, School of Public Health, University of Texas Health Science Center at Houston, Houston, TX, US

1200 Pressler St, Suite E405, Houston, TX, US 77584

Phone: +1 713-500-9285; email: [bing.yu@uth.tmc.edu](mailto:bing.yu@uth.tmc.edu)

SUPPLEMENTARY FILES

**Supplementary Text 1.** Definition of MI by each COMETS cohort, and definition of the covariables used to adjust the statistical models.

**Supplementary Text 2.** STROBE checklist.

**Supplementary Table 1.** Metabolites significantly associated (meta-analysis FDR < 0.05) with incident MI. TE and SE refer to the estimated overall treatment effect and standard error, respectively.

**Supplementary Table 2.** Literature references for the metabolites previously associated with any cardiac diseases, and the super- and sub-pathways for metabolites associated with incident MI. For the metabolites that did not remain significant after further adjusting the meta-analyses for prevalent hypertension, dyslipidaemia and type-2 diabetes, references showing their associations with any of these 3 conditions are indicated.

**Supplementary Table 3.** Results of the random effect inverse-variance meta-analysis performed in the MI-associated metabolites (meta-analysis FDR < 0.05) based on the results from the fixed effect inverse-variance meta-analysis. TE and SE refer to the estimated overall treatment effect and standard error, respectively.

**Supplementary Table 4.** Meta-analysis results from the 56 metabolites significantly associated with incident MI when the analyses were run excluding the cohorts in which MI was assessed by self-reported questionnaires (TwinsUK and ET2DS). TE and SE refer to the estimated overall treatment effect and standard error, respectively.

**Supplementary Table 5.** Meta-analysis results from the 56 metabolites significantly associated with incident MI when the models were further adjusted for prevalent hypertension, prevalent type-2 diabetes, and prevalent dyslipidaemia. Significant associations are marked in red. TE and SE refer to the estimated overall treatment effect and standard error, respectively.

**Supplementary Table 6.** Meta-analysis results from the 56 metabolites significantly associated with incident MI when the models were stratified by race (White individuals and Black individuals). Significant associations are marked in red. TE and SE refer to the estimated overall treatment effect and standard error, respectively.

**Supplementary Table 7.** Metabolites associated (meta-analysis nominal p-value < 0.05) with prevalent MI, and that are also significantly associated with incident MI (meta-analysis FDR < 0.05). TE and SE refer to estimated overall treatment effect and standard error, respectively.

**Supplementary Table 8** Enrichment pathway analysis results showing all the identified pathways. ‘Total’ indicates the number of metabolites that are involved in each pathway, whereas ‘Hits’ indicates the number of metabolites associated with incident MI that is present in each pathway.
