# Supplementary material for: Predictive metabolites for incident myocardial infarction: a two-step meta-analysis of individual patient data from six cohorts comprising 7897 individuals from the COnsortium of METabolomics Studies
Source: Cardiovasc Res. 2023 Sep 14;119(17):2743–54. doi: 10.1093/cvr/cvad147 (PMC10757581; doi:10.1093/cvr/cvad147)
Supplement: cvad147_Supplementary_Data [file cvad147_supplementary_data.zip › suppl_text1.docx]

**Supplementary Text 1. Definition of MI by each COMETS cohort, and definition of the covariables used to adjust the statistical models.**

MI was defined by each cohort as followed:

- ARIC: MI was assessed by hospital records and echocardiograms at follow-up visits, and by participants’ medical histories and electrocardiograms administered at baseline visits.

- ET2DS: International Classification of Diseases (ICD) codes 121-123 were used for hospitalization and death records. Chest pain questionnaires, general practitioner records, and self-report questionnaires were also used to assist in identification.

- GDM: The patient’s electronic medical records were reviewed and consider MI whether MI diagnosis was given by a cardiologist based on clinical evidence and complementary tests.

- HABC: Each participant was contacted every 6 months to query hospitalizations or major outpatient procedures. If participants could not be reached, information was ascertained from proxies. Records from all overnight hospitalizations were obtained and reviewed for incident MI. MI diagnoses were adjudicated by physicians at clinical sites from hospitalization and/or death records. The underlying and contributing causes of death were obtained from death certificates and including the adjudication.

- TwinsUK: MI was self-reported in questionnaires.

- WHI: In the WHI, the endpoint included MI or death due to CHD, and all events were confirmed by medical records and adjudication by a physician.

- KORA: MI were identified via the KORA Augsburg coronary event registry or through questionnaires for participants residing outside the study area ^59^.

MI prevalence and incidence, and the covariables were coded identically across the cohorts as indicated below:

- Prevalent MI: A given person had already suffered from MI at the time of the blood sample/metabolomics profiling (Prevalent MI = 1; dichotomous variable).

- Incident MI: A given person suffered from MI after the blood sample/metabolomics profiling (Incident MI = 1; dichotomous variable).

- age baseline: age (years) of a subject at baseline timepoint.

- age follow-up: age (years) of a subject at follow-up timepoint.

- gender: dichotomous variable (1 or 0):

- 1 = male

- 0 = female

- BMI: body mass index (kg/m^2^) of a subject at baseline timepoint.

- Smoking status: categorical variable with 3 levels:

- 0 = never smoker

- 1 = former smoker

- 2 = current smoker

- Race: categorical variable indicating the ancestry:

- 0 = White/European ancestry

- 1 = Non-European ancestry

-Education level: categorical variable with 4 levels:

- 0 = did not complete high school

- 1 = completed high school

- 2 = post-high school training/ some college

- 3 = completed college

- Alcohol consumption: categorical variable with 4 levels:

- 0 = zero alcohol intake

- 1 = <0, 15] g/day

- 2 = <15, 30] g/day

- 3 = >30 g/day

- Physical activity level: categorical variable with 3 levels:

- 0 = low

- 1 = medium or missing

- 2 = high

-Prevalent type-2 diabetes: dichotomous variable (1 or 0):

- 0 = Type-2 diabetes had not been diagnosed by the time of the blood sample/metabolomics profiling (baseline)

- 1 = Type-2 diabetes had been diagnosed by the time of the blood sample/metabolomics profiling (baseline)

- Prevalent hypertension (defined as systolic blood pressure>140 mmHg or diastolic blood pressure >90 mmHg or taking hypertension-lowering medications or diagnosed by the doctor as having hypertension at baseline): dichotomous variable (1 or 0):

- 0 = Hypertension had not been diagnosed by the time of the blood sample/metabolomics profiling (baseline)

- 1 = Hypertension had been diagnosed by the time of the blood sample/metabolomics profiling (baseline)

- Prevalent dyslipidaemia (defined as high levels of total cholesterol (>240 mg/dL) or high levels of triglycerides (≥ 500 mg/dL) or low levels of HDL cholesterol (≤40 mg/dL): dichotomous variable (1 or 0):

- 0 = Dyslipidaemia had not been diagnosed by the time of the blood sample/metabolomics profiling (baseline)

- 1 = Dyslipidaemia had been diagnosed by the time of the blood sample/metabolomics profiling (baseline)
